# Captivating Colors, Crucial Roles: Astaxanthin’s Antioxidant Impact on Fish Oxidative Stress and Reproductive Performance

**DOI:** 10.3390/ani13213357

**Published:** 2023-10-29

**Authors:** Yauheni Shastak, Wolf Pelletier

**Affiliations:** Nutrition & Health Division, BASF SE, 67063 Ludwigshafen am Rhein, Germany

**Keywords:** astaxanthin, fish, nutrition, antioxidant, reproduction, oxidative stress markers, fish health, ecological sustainability

## Abstract

**Simple Summary:**

Oxidative stress represents a significant threat to fish health, resulting in cellular damage and a host of health challenges. Astaxanthin, a powerful antioxidant, plays a pivotal role in reducing the reactivity of harmful reactive oxygen species and safeguarding crucial biomolecules within fish. Beyond its antioxidant properties, astaxanthin also regulates redox-sensitive signaling pathways, potentially enhancing fish health and well-being. Oxidative stress can impact fish reproduction, and astaxanthin supplementation has demonstrated promise in bolstering reproductive performance, encompassing improvements in egg quality and hormone levels. Furthermore, the integration of astaxanthin into aquaculture practices holds substantial potential for enhancing fish health and overall productivity, aligning with the sustainability goals of the aquaculture industry.

**Abstract:**

Fish, constantly exposed to environmental stressors due to their aquatic habitat and high metabolic rates, are susceptible to oxidative stress. This review examines the interplay between oxidative stress and fish reproduction, emphasizing the potent antioxidant properties of astaxanthin. Our primary objective is to highlight astaxanthin’s role in mitigating oxidative stress during critical reproductive stages, leading to improved gamete quality, ovary development, and hormone levels. We also explore its practical applications in aquaculture, including enhanced pigmentation and overall fish health. We conducted a comprehensive literature review, analyzing studies on astaxanthin’s antioxidant properties and its impact on fish reproduction. Astaxanthin, a carotenoid pigment, effectively combats reactive oxygen species, inhibiting lipid peroxidation and maintaining membrane integrity. It significantly enhances reproductive success in fish and improves overall fish health in aquaculture settings. This review reveals astaxanthin’s multifaceted benefits in fish health and reproduction, offering economic advantages in aquaculture. Future research should delve into species-specific responses, optimal dosages, and the long-term effects of astaxanthin supplementation to inform sustainable aquaculture strategies.

## 1. Introduction

In the domain of aquatic ecosystems, prioritizing the vitality and thriving of fish populations takes precedence. A pivotal determinant influencing fish well-being is oxidative stress [1], a condition characterized by the disproportion between the generation of detrimental reactive oxygen species (ROS) and an organism’s capacity to mitigate their injurious consequences through antioxidants [2]. This intricate equilibrium plays a vital role in upholding the comprehensive health and efficiency of fish, underscoring the significance of researching antioxidants as a fundamental constituent of fisheries science and aquaculture management [3,4].

One antioxidant that has attracted considerable attention recently is astaxanthin—a naturally occurring carotenoid pigment, also attainable through synthetic means, responsible for the vivid red and orange hues manifested in diverse aquatic organisms, such as salmon, shrimp, and crayfish [5,6]. Natural sources of astaxanthin primarily include specific microalgae species and red yeast. These organisms produce astaxanthin as a defense mechanism against environmental stressors [7]. Commercially, astaxanthin extraction from these microorganisms employs various techniques, such as supercritical CO_2_ extraction or solvent extraction [8,9]. In contrast, synthetic production involves chemical synthesis using specific precursors and controlled reactions in industrial settings [10]. The chemical synthesis of astaxanthin typically entails the Wittig reaction, which necessitates the use of the precursor β-ionone and a carbonyl compound [11].

Astaxanthin is a prevalent pigment employed extensively within the field of aquaculture [10]. Beyond its aesthetic appeal, astaxanthin has emerged as a powerful player in promoting fish health and longevity through its remarkable antioxidant properties [5,10]. These properties allow astaxanthin to neutralize ROS and mitigate the detrimental impacts of oxidative stress, thereby safeguarding cellular integrity and essential physiological processes [12].

Astaxanthin has earned the moniker “super vitamin E” due to its exceptional free radical scavenging capabilities, which surpass those of other carotenoids including β-carotene, canthaxanthin, lutein, and zeaxanthin, as well as vitamins C and E [13]. The in vitro antioxidant potential of astaxanthin demonstrated a significant superiority, with a potency that surpassed zeaxanthin, lutein, canthaxanthin, and β-carotene by a factor of 10 and even exceeded α-tocopherol by a notable 100-fold in neutralizing toxic ROS without forming pro-oxidants [14]. In comparison to vitamin C, astaxanthin exhibited a potency that was 6000 times greater [15].

Vibrant coloration in fish frequently indicates robust health and optimal physiological performance [16]. Consequently, scientists and conservation experts employ this coloration phenomenon as a visual indicator for tracking the vitality of aquaculture communities and gauging the holistic ecological balance within aquatic environments [17]. The detection of astaxanthin, along with its influence on coloration, presents a valuable mechanism for comprehending the intricate interactions among environmental variables, fish well-being, and the intricate dynamics of ecosystems [18]. 

Recent research has highlighted the complex relationship between oxidative stress and reproductive processes in fish biology [19]. Oxidative stress can negatively affect gamete quality, leading to compromised structural and functional integrity in sperm and ova [20,21,22]. This can result in reduced fertilization success and developmental anomalies in the offspring. The central player in this interplay is nuclear factor erythroid-2 related factor 2 (Nrf2), a transcription factor regulating antioxidant gene expression that can become disrupted under prolonged oxidative stress, compromising antioxidant defenses [23]. Oxidative stress can also disrupt endocrine pathways related to fish reproduction and is exacerbated by environmental stressors such as pollutants and temperature fluctuations [24,25,26,27]. Understanding these dynamics has implications for fish conservation and aquaculture, including the development of biomarkers and antioxidant-based interventions to improve reproductive success.

The primary goal of this paper is to offer an extensive examination of the current knowledge concerning the interplay between astaxanthin and oxidative stress, as well as the intricate connections linking astaxanthin, oxidative stress, and fish reproductive performance within aquaculture production. Specifically, this review will concentrate on the following key facets:

1. Oxidative stress and fish health
2. Mechanisms of astaxanthin as an antioxidant
3. Astaxanthin and oxidative stress regulation in fish
4. Reproductive performance and oxidative stress: interplay and implications
5. Astaxanthin’s influence on fish reproductive performance
6. Practical applications of astaxanthin in aquaculture
7. Future directions and research gaps

By thoroughly analyzing these seven topics, this review aims to provide additional insights into the potential advantages of using astaxanthin as a nutritional approach to enhance fish health and productivity. Furthermore, it intends to highlight areas in need of further investigation, thus contributing to the advancement of knowledge in this domain.

## 2. Oxidative Stress and Fish Health

Oxidative stress holds significant ramifications for the well-being of aquatic organisms, with fish exhibiting heightened vulnerability to its perturbations [28,29]. ROS, encompassing entities such as superoxide anions, hydrogen peroxide, and hydroxyl radicals, emerge as natural byproducts of cellular metabolic processes [2]. However, when their generation surpasses the buffering capabilities of antioxidant defenses, the resultant oxidative stress leads to cellular impairment, DNA mutations, and disturbances in pivotal biomolecules [2]. Collectively, these processes contribute to an array of health challenges in fish [30]. 

The predominant contributor to ROS is the mitochondrial electron transport chain, an essential component of cellular respiration [31,32,33]. Furthermore, internal mechanisms like peroxisomal reactions and inflammatory responses can trigger the production of ROS [34,35,36]. Specifically, peroxisomal reactions can trigger ROS production as a byproduct of processes like fatty acid oxidation and detoxification, generating hydrogen peroxide and superoxide anions [37,38]. Inflammatory responses activate immune cells, such as neutrophils and macrophages, which produce ROS through enzymes like NADPH oxidase during their antimicrobial defense mechanisms, contributing to ROS production during inflammation [39]. External elements, including exposure to pollutants, heavy metals, and ultraviolet radiation, can intensify ROS generation [40,41]. The cumulative effect can overwhelm the antioxidant defense system, encompassing both enzymatic antioxidants like superoxide dismutase, catalase, and glutathione peroxidase, as well as non-enzymatic antioxidants such as vitamins C and E, glutathione, and carotenoids [42,43].

The effects of oxidative stress propagate through various physiological processes in fish, influencing multiple facets of their biology [44]. One of the most pivotal domains influenced is the immune system [34]. ROS, while critical in mounting immune responses against pathogens [45], can paradoxically inhibit immune functioning when their levels surge uncontrollably, rendering fish more susceptible to infections [34,46]. The impact of oxidative stress further resonates in fish reproduction, exerting influence over sperm quality and egg viability, potentially undermining reproductive success [20,47]. Moreover, oxidative stress interplays with fish growth and development [48]. The balance between ROS production and antioxidant defenses is particularly precarious during periods of rapid growth, as oxidative stress can slow down the normal process of growth and development [49].

Beyond physiological realms, oxidative stress infiltrates the behavioral patterns and adaptability of fish. Prolonged exposure to oxidative stress can trigger alterations in behavior, manifesting as reduced swimming performance and compromised responses to predator threats [50,51,52]. Physiological stress resulting from oxidative imbalance also reverberates through fish energy metabolism. Damage inflicted upon mitochondria—the cellular powerhouses—by ROS can lead to diminished energy production and compromised endurance in fish, a predicament particularly worrisome for migratory species dependent on sustained energy for extensive journeys [53,54,55]. 

**Figure 1:**
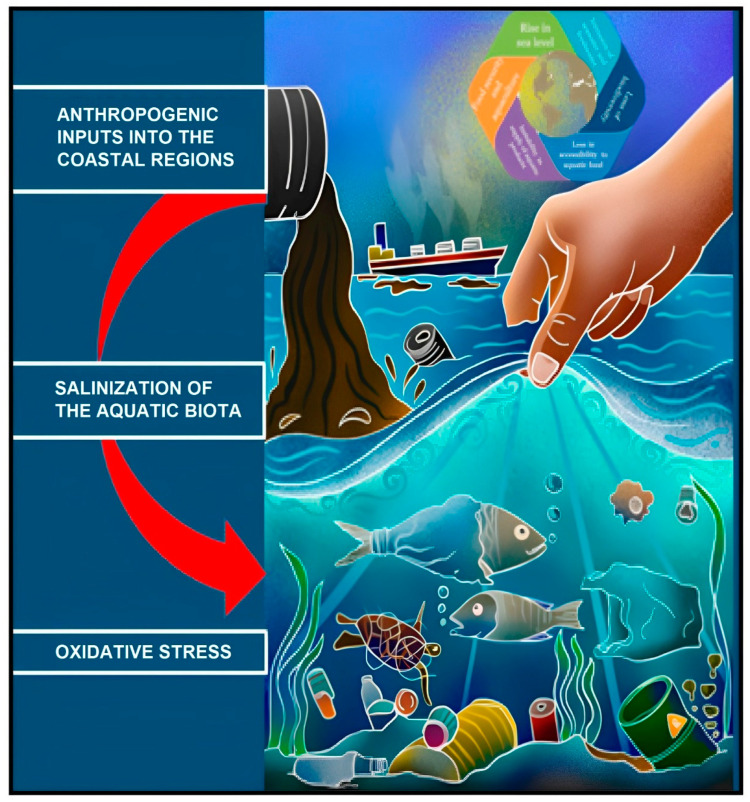
An example of anthropogenic impact: amplifying physicochemical parameters and climate-related salinity fluctuations affecting the oxidative health of coastal aquatic organisms [56]. In coastal and inland areas, there is a persistent and concerning rise in groundwater salinity. This increase is primarily attributed to the continual depletion of groundwater resources, coupled with the constant dissolution of salts from the Earth’s surface and the introduction of heat-trapping pollutants from human activities.

The increasing anthropogenic pressures on aquatic ecosystems, coupled with genetic selection aimed at achieving rapid growth and efficient fish muscle deposition under commercial rearing conditions, have contributed to a substantial rise in oxidative stress within ichthyoid populations [56,57,58,59,60] (Figure 1). Pollution, climate change, and habitat degradation converge to amplify ROS production in aquatic environments [60]. Heavy metal contamination in water bodies, for instance, directly augments ROS levels within fish due to metal-catalyzed oxidative reactions [40]. Elevated water temperatures linked to climate change exacerbate oxidative stress by stimulating metabolic rates and intensifying ROS production [61]. As aquatic ecosystems endure these perturbations, comprehending the interplay between oxidative stress and fish health becomes a pressing concern.

Even within controlled environments such as enclosed fish farms or recirculating aquaculture systems that meticulously manage water quality and temperatures, the possibility of oxidative stress persists [62]. This potential arises from factors including unbalanced or insufficient diets, the presence of antinutrients in feeds, challenges associated with handling and transportation throughout various fish production stages, elevated stocking densities, suboptimal management practices, the application of specific medications, the use of oxidative disinfectants, and the influence of genetic predispositions [63,64,65]. Some common oxidative stressors for fish and their permissible limits are shown in Table 1.

## 3. Mechanisms of Astaxanthin as an Antioxidant

In recent years, there has been a growing interest in astaxanthin due to its recognized antioxidative properties, particularly within the context of fish physiology [5,76]. Fish, being aquatic organisms, are continuously exposed to a variety of environmental stressors. To mitigate the potential damage inflicted by oxidative stress, fish have developed complex antioxidant defense mechanisms. Within this framework, the effectiveness of astaxanthin as a robust and multifaceted antioxidant is notable [5]. The antioxidant attributes of astaxanthin are intricately linked to its underlying chemical configuration [77]. 

### 3.1. ROS Scavenging Mechanism

**Figure 2:**
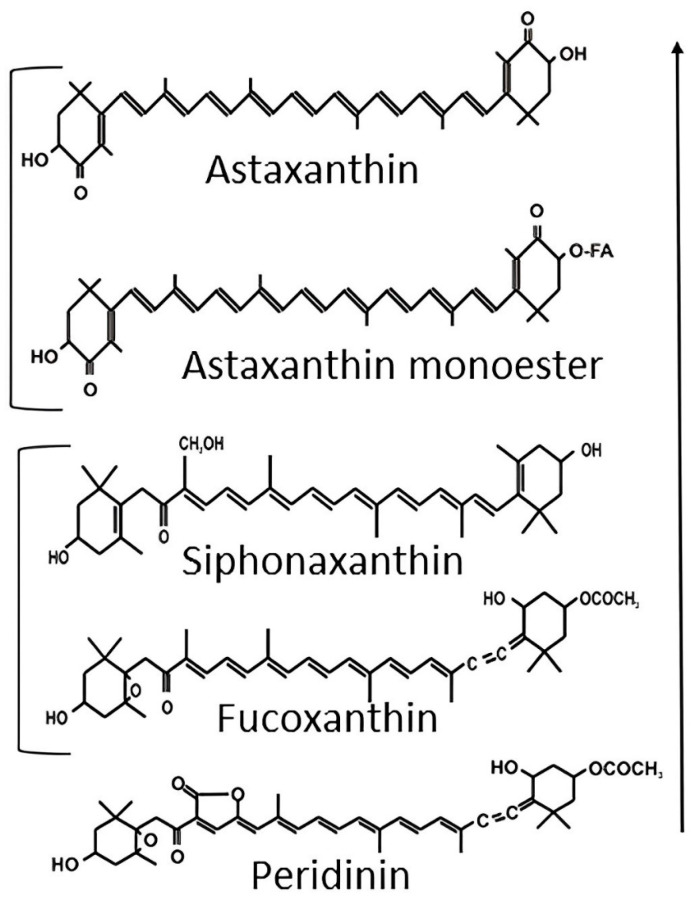
Comparing the ^1^O_2_ quenching activities of some carotenoids. Arrows represent increasing activity, while brackets denote similar activity [78].

Astaxanthin’s structural features, including its conjugated double bond system and extended polyene arrangement, create an optimal framework for electron distribution, enabling effective modulation of ROS activity [78] (Figure 2). The conjugated double bonds also give astaxanthin its characteristic red color, which is often visible in the flesh of certain fish species, such as salmon and trout [79]. Notably, the hydroxyl and keto functional groups within astaxanthin’s molecular structure play a crucial role in facilitating electron transfer to unstable ROS, thereby reducing their reactivity and protecting biomolecules such as lipids, proteins, and nucleic acids from oxidative damage [80]. Importantly, astaxanthin’s potential to neutralize ROS might surpass that of both β-carotene and α-tocopherol [81]. 

**Figure 3:**
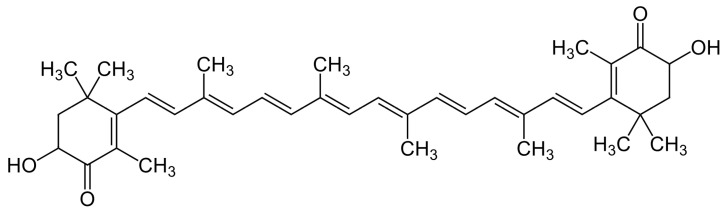
Structural formula for astaxanthin.

In the context of photosynthesis, singlet oxygen (^1^O_2_) can arise from light-interacting chlorophyll molecules in plants [82]. Similarly, in fish, ^1^O_2_—a highly reactive and toxic form of ROS—could be generated naturally during cellular metabolism [83]. This reactive species, ^1^O_2_, possesses the ability to induce oxidative stress within ichthyoid cells [84]. Similar to its interactions with other ROS, the efficient quenching of ^1^O_2_ by astaxanthin can be attributed to its configuration of conjugated double bonds. Additionally, the presence of terminal ionone rings at both ends of the molecule also contributes significantly to this process (Figure 3). These structural features facilitate energy transfer between the excited ^1^O_2_ and the astaxanthin molecule [85]. Consequently, this energy transfer dissipates the excess energy of ^1^O_2_, rendering it inactive and preventing detrimental interactions with cellular components. In this manner, astaxanthin’s capacity to quench ^1^O_2_ contributes to its role as a robust antioxidant in fish tissues [86]. 

### 3.2. Lipid Peroxidation Inhibition

**Figure 4:**
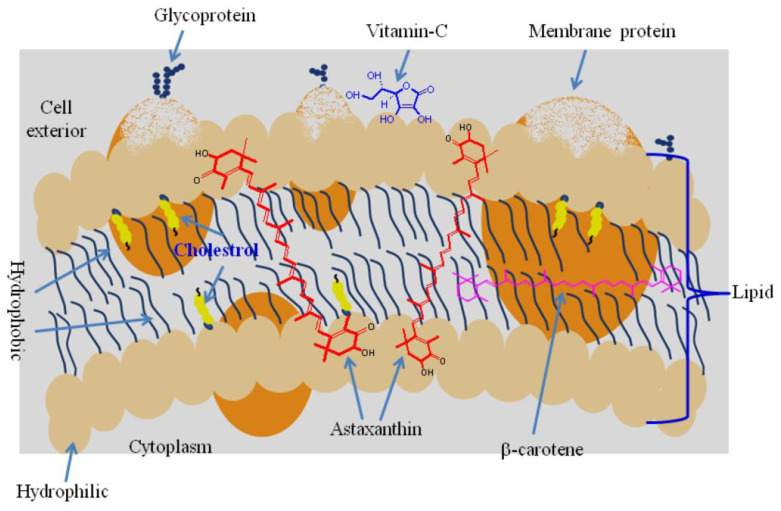
Position of astaxanthin in the cell membrane [79].

Lipid peroxidation is a detrimental biochemical process involving the oxidative deterioration of polyunsaturated fatty acids within cellular membranes, leading to the potential disruption of membrane structural integrity and the compromise of vital cellular functions [87]. Astaxanthin demonstrates effectiveness in mitigating lipid peroxidation due to its lipophilic nature, which facilitates its strategic integration into cellular membranes [79]. Its molecular arrangement, characterized by a sequence of linear, polar, nonpolar, and polar components, allows precise integration within the membrane’s lipid bilayer, spanning its entirety (see Figure 4). As astaxanthin is incorporated into the lipid bilayers, its polar segments extend into the surrounding aqueous environment, while its hydrophobic region interacts with the hydrocarbon chains of membrane lipids [88,89]. This molecular disposition hinders the propagation of lipid peroxidation reactions by quenching lipid radicals and stabilizing the lipid bilayer structure [90,91]. In this configuration, astaxanthin possesses the capacity to intercept reactive molecular species within the lipid core of the membrane and at its interfaces with the surrounding aqueous phase [92]. Moreover, astaxanthin’s antioxidant attributes extend to synergistic interactions with other antioxidants, such as vitamin E, fostering a concerted defense against lipid peroxidation [93,94]. Specifically, astaxanthin and vitamin E synergize through the possible regeneration of vitamin E by astaxanthin [95]. 

## 4. Astaxanthin and Oxidative Stress Regulation in Fish

Over the years, a substantial body of research has emerged, illuminating the capacity of astaxanthin to influence antioxidant enzyme activity and gene expression within fish species [5,23,96,97]. Astaxanthin’s potential to modulate these vital aspects of cellular defense mechanisms has garnered significant attention due to its implications for fish health, aquaculture practices, and the broader understanding of oxidative stress regulation in aquatic organisms [98,99]. 

Hassanzadeh et al. [97]. conducted a study to examine the potential effects of incorporating synthetic astaxanthin into the diet of rainbow trout. The researchers assessed two different dietary levels: 0.5 and 2 g per kg of feed. Over a duration of 60 days, the fish were fed with these experimental diets and subsequently exposed to paraquat, a known oxidative stress inducer. The results revealed that the inclusion of astaxanthin in the diets led to noticeable improvements (*p* < 0.05) in growth rates [97]. Additionally, the fish that received astaxanthin-supplemented diets exhibited reduced levels of protein and lipid oxidation, as well as lower peroxide values compared to the control group (*p* < 0.05) that was exposed to paraquat alone. Furthermore, the rainbow trout fed with astaxanthin displayed an increased expression of genes linked to antioxidant defense mechanisms (*p* < 0.05). This suggests that astaxanthin likely contributed to enhancing the overall antioxidant system of the fish.

In their 2023 research, Shabanzadeh and colleagues [23] conducted a comprehensive investigation into the impact of commercial synthetic astaxanthin on various parameters in rainbow trout when faced with diazinon exposure. The study encompassed a dietary regimen where fish were administered varying concentrations of astaxanthin (0.0, 0.5, 2.0, and 5.0 g/kg) for a span of 60 days, followed by a subsequent diazinon challenge. The outcomes demonstrated significant improvements (*p* < 0.05) in growth patterns across all astaxanthin-enriched diets as compared to the control group, which did not receive supplementary astaxanthin. The investigation further probed the serum antioxidant status of the fish. The findings exhibited that those fish that were fed diets enriched with astaxanthin, especially at the 2.0 and 5.0 g/kg levels, displayed enhanced serum antioxidant levels (*p* < 0.05). This enhancement was evident through a decrease in malondialdehyde levels, a well-established marker of oxidative stress, and an increase in overall antioxidant activity. Central to the study was the analysis of gene expression patterns. The researchers documented a substantial up-regulation of vital antioxidant-related genes in the kidneys and liver of fish that received astaxanthin supplementation (*p* < 0.05), particularly in the highest dosage group. The genes included superoxide dismutase, catalase, glutathione peroxidase, glutathione S-transferase, and Nrf2. This observation suggests that astaxanthin may have the potential to activate these genes, bolstering the fish’s ability to counteract the oxidative stress prompted by diazinon exposure.

**Figure 5:**
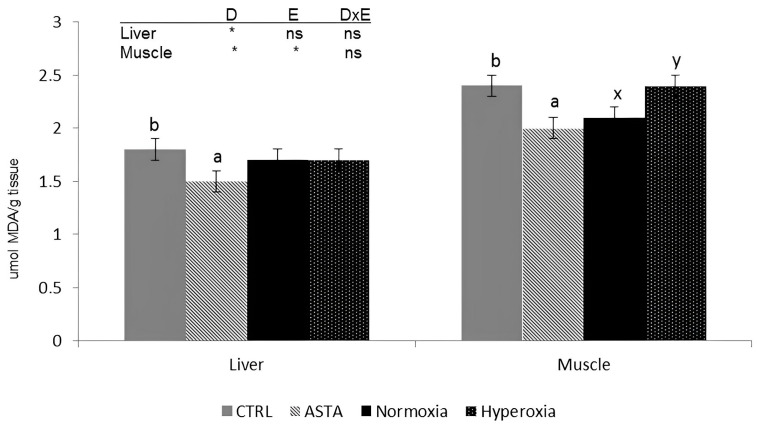
Liver and muscle thiobarbituric acid-reactive substances (TBARS) (*n* = 12) of rainbow trout without astaxanthin supplementation (CTRL) and synthetic astaxanthin-fed (ASTA) rainbow trout exposed to a normoxic environment (12 weeks) and episodic hyperoxic environment (1 week) [96]. Values are presented as means ± SEM. Different superscript letters (^a,b^ for diet factor and ^x,y^ for environment factor) within a tissue denote significant differences between factors determined by two-way ANOVA (*p* < 0.05). D, diet factor; E, environment factor; DxE, interaction between diet and environment; MDA = malondialdehyde; * *p* < 0.05; ns not significant.

In 2019, Kalinowski et al. [96]. conducted a 13-week trial involving juvenile rainbow trout to investigate the impacts of two diets: a control diet and another enriched with 100 mg/kg of synthetic astaxanthin. The researchers employed intermittent hyperoxia exposure to induce stress responses. The introduction of astaxanthin supplementation demonstrated notable effects on reducing oxidative stress. This was evident through a decrease in thiobarbituric acid-reactive substances, indicative of lipid peroxidation products in muscle and liver cells (Figure 5). While hyperoxia triggered a decline in liver antioxidant enzyme activities, the inclusion of astaxanthin led to increased glutathione reductase activity, corroborated by higher mRNA levels (*p* < 0.05). Moreover, astaxanthin supplementation improved the ratio of reduced glutathione to oxidized glutathione (*p* < 0.05). This improvement was attributed to enhanced glutathione recycling and de novo synthesis, facilitated by the up-regulation of related enzyme mRNA expression. Additionally, the astaxanthin-enriched diet prompted elevated mRNA expression of liver glucokinase and glucose-6-phosphate dehydrogenase, suggesting potential activation of the NADPH-producing pentose phosphate pathway, which counteracts oxidative stress. In essence, this study highlights the potential of dietary astaxanthin to alleviate oxidative stress in the context of hyperoxia-induced conditions.

Zhu et al. [5] conducted an investigation into the influence of astaxanthin on the antioxidative function and associated traits of coral trout. The research employed four distinct diets (designated as A0, A1, A2, and A3) containing varying levels of astaxanthin (0, 0.05, 0.1, and 0.2 g/kg) sourced from *Haematococcus pluvialis* powder. Over the course of an 8-week feeding trial, the supplementation of astaxanthin demonstrated no statistically significant (*p* > 0.05) impacts on the growth of coral trout. However, a notable enhancement in antioxidant activity was observed within the A2 diet group, characterized by elevated levels of catalase, superoxide dismutase, and glutathione peroxidase activities, along with heightened total antioxidant capacity levels present in both the serum and liver (*p* < 0.05). Subsequent to a challenge involving *Vibrio harveyi*, the A2 diet group exhibited increased levels of serum and liver acid phosphatase, lysozyme activities, and complement contents (*p* < 0.05). Furthermore, the expression of genes in the liver associated with antioxidant enzymes, immune responses, and defense mechanisms was found to be up-regulated within the A2 group (*p* < 0.05). Remarkably, the A2 diet group displayed a notably improved survival rate following exposure to the *V. harveyi* challenge (*p* < 0.05). To conclude, the inclusion of 1.0 g/kg of *H. pluvialis* powder rich in astaxanthin (equivalent to 0.091 g/kg astaxanthin) resulted in the augmentation of antioxidant capacity, immune response, and resistance against the *V. harveyi* challenge among coral trout.

Xie et al. [98] conducted a comprehensive investigation into the influence of astaxanthin on the growth performance and antioxidant functions of golden pompano (*Trachinotus ovatus*) through both in vivo and in vitro experiments. During the in vivo phase, fish were subjected to diets with or without astaxanthin supplementation (0 and 200 mg/kg) over a span of 6 weeks. The inclusion of astaxanthin in the diet resulted in statistically significant enhancements in weight gain and specific growth rate, accompanied by a notable reduction in the feed conversion ratio within the astaxanthin-supplemented group (*p* < 0.05). This supplementation also triggered elevated hepatic total antioxidant capacity and augmented levels of reduced glutathione within the astaxanthin-fed cohort (Table 2). Conversely, there was a decrease observed in superoxide dismutase levels due to the astaxanthin supplementation. The in vitro aspect of this study involved the exposure of isolated hepatopancreas cells to oxidative stress instigated by hydrogen peroxide (H_2_O_2_). The viability of these cells exhibited a decline upon H_2_O_2_ treatment, which was effectively counteracted by the introduction of astaxanthin supplementation (*p* < 0.05). Oxidative stress induced a reduction in both total antioxidant capacity and reduced glutathione levels, while concurrently leading to an elevation in malondialdehyde concentrations. Nevertheless, the addition of astaxanthin served to ameliorate these effects and resulted in a reduction of malondialdehyde levels (*p* < 0.05). Collectively, these findings strongly indicate that astaxanthin plays a pivotal role in enhancing hepatic antioxidant capacity, both in vivo and in vitro, by effectively mitigating the deleterious impacts of ROS-induced damage.

In addition to influencing the activity of antioxidant enzymes and gene expression, astaxanthin operates through complex interactions with redox-sensitive signaling pathways in fish [99]. The presence of oxidative stress not only jeopardizes cellular integrity but also plays a crucial role in cellular signaling, impacting a range of physiological processes. Astaxanthin’s capacity to regulate redox-sensitive pathways assumes a dual role: safeguarding against oxidative damage and participating in signaling cascades [77,100]. Remarkably, the impact of astaxanthin on the nuclear factor-erythroid 2-related factor 2 (Nrf2) pathway has attracted considerable attention [23]. The Nrf2 pathway functions as a central overseer of the cellular antioxidant response, coordinating the activation of various genes responsible for cellular protection against oxidative stress [101,102]. Astaxanthin has demonstrated the ability to stimulate the Nrf2 pathway, leading to an increase in the expression of antioxidant enzymes [23]. Furthermore, astaxanthin’s interactions with other signaling pathways, such as mitogen-activated protein kinases (MAPKs) and nuclear factor-kappa B (NF-κB), underscore its potential to regulate broader cellular responses that extend beyond antioxidative defenses [103,104]. 

Astaxanthin’s role in modulating oxidative stress in fish presents promising implications for both aquaculture and environmental applications. In the realm of aquaculture, where stressors encompass a spectrum from physical handling and transportation to suboptimal water conditions, the addition of astaxanthin holds potential as a strategy to enhance fish resilience [98,105]. Through its capacity to augment antioxidant enzyme activity and influence gene expression, astaxanthin supplementation may offer a means to alleviate the adverse effects of stress on fish well-being and overall performance [106]. Furthermore, investigating how astaxanthin interacts with redox-sensitive signaling pathways could uncover innovative avenues for advancing aquaculture methodologies, fostering sustainable fish production, and minimizing ecological repercussions. Table 3 presents the effects of astaxanthin on oxidative status and growth across various fish species.

## 5. Reproductive Performance and Oxidative Stress: Interplay and Implications

The intricate interplay between oxidative stress and reproductive processes has emerged as a captivating avenue of research in recent years [19]. In the context of fish reproductive biology, this interaction is particularly intriguing due to its potential implications for gamete quality and fertility, crucial components of successful reproduction [47,61]. The exploration of the underlying mechanisms governing the relationship between oxidative stress and reproductive challenges in fish not only advances our comprehension of their physiological responses but also offers novel insights applicable to conservation and aquaculture practices.

A notable facet of the interplay between oxidative stress and fish reproductive performance lies in the influence of ROS on gamete quality. Sperm and ova are exquisitely sensitive to oxidative damage due to their lipid-rich composition and limited antioxidant defenses [20,21,22]. Oxidative stress can trigger lipid peroxidation, protein modifications, and DNA damage in these gametes, culminating in compromised structural integrity and functional aptitude [61]. The prevalence of mitochondria in sperm renders them especially vulnerable to ROS-mediated impairment, resulting in diminished motility and viability [111]. Additionally, oxidative stress-induced DNA damage in sperm has been correlated with reduced fertilization success and an increased occurrence of developmental anomalies in offspring [47,112,113]. Likewise, oxidative stress can disturb oocyte maturation, hinder fertilization, and undermine early embryonic development [20,114]. These deleterious effects on gamete quality underscore the paramount significance of upholding redox homeostasis to ensure successful fertilization and embryogenesis in fish.

The complex mechanisms connecting oxidative stress to reproductive challenges are intricate and multifaceted. At the heart of this interplay lies Nrf2, a transcription factor with a pivotal role in regulating antioxidant gene expression [23]. In the context of oxidative stress, Nrf2 becomes activated, driving the elevation of antioxidant enzymes like superoxide dismutase, catalase, and glutathione peroxidase [115,116,117]. Nonetheless, prolonged oxidative stress could potentially disrupt Nrf2 signaling, leading to compromised antioxidant functionality and heightened vulnerability to oxidative harm in gametes [118,119]. Furthermore, the activation of Nrf2 might shift resources away from other vital physiological processes, exerting influences on reproductive allocation and overall success [120,121]. 

Moreover, oxidative stress has the potential to disturb endocrine pathways integral to fish reproduction, such as the hypothalamus-pituitary-gonadal axis. This disruption can interfere with hormone synthesis, release, and receptor interactions, thereby impacting fish reproductive functions [24,25,26,27]. The consequences of these disruptions might manifest as irregularities in gametogenesis, spawning behavior, and hormone-mediated physiological adjustments, ultimately leading to compromised reproductive achievement.

An often-understated aspect concerning the interplay linking oxidative stress and fish reproductive mechanisms involves the pivotal contribution of environmental stressors. Human-driven activities have resulted in the pervasive contamination of aquatic ecosystems with an array of pollutants, heavy metals, and other noxious agents, which possess the potential to amplify oxidative stress within fish organisms [122,123]. Prolonged exposure to these stress-inducing factors has the capacity to disrupt the intricate equilibrium governing the generation of ROS and the counteracting mechanisms of antioxidant defenses. Consequently, this disruption can heighten the influence on the quality of gametes and overall fertility [124,125]. Additionally, the ever-changing circumstances of the environment, including temperature fluctuations and oxygen availability variations, wield the capability to exert an impact on the redox status of fish organisms, thereby exacerbating the redox imbalance during pivotal reproductive phases [61,126,127,128]. These complex interplays involving oxidative stress, environmental stressors, and reproductive processes underscore the imperative of comprehensively grasping the realm of fish reproductive biology within the context of their native habitats.

The implications of the interrelationship between oxidative stress and fish reproductive performance extend beyond the realm of basic biology. In the scope of conservation, this interplay can shed light on the potential consequences of habitat degradation and pollution on fish populations. By unraveling the underlying mechanistic connections linking oxidative stress to reproductive hurdles, researchers can forge biomarkers for evaluating the reproductive well-being of fish communities and projecting their susceptibility to environmental stressors [129]. Correspondingly, within the field of aquaculture, comprehending the ramifications of oxidative stress on gamete quality and fertility assumes pivotal importance in the refinement of breeding protocols and the innovation of antioxidant-centered interventions. Manipulating the dietary intake of antioxidants or employing antioxidant supplementation strategies could potentially mitigate the negative effects of oxidative stress on reproductive performance, thereby enhancing the success of aquaculture practices [130,131,132]. 

## 6. Astaxanthin’s Influence on Fish Reproductive Performance

**Figure 6:**
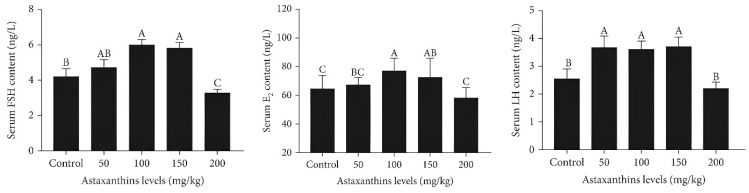
Serum hormone levels in Nile tilapia fed with diets containing astaxanthin were at different levels for 60 days [133]. Sixteen samples from each group were analyzed. Different uppercase letters indicate significant differences among different groups (Duncan’s multiple range test, *p* < 0.05). LH = luteinizing hormone; FSH = follicle-stimulating hormone; E_2_ = estradiol.

Recent scientific investigations delving into the effects of astaxanthin supplementation on the reproductive outcomes of fish have provided valuable insights, shedding light on the potential role of this carotenoid in bolstering various dimensions of ichthyoid reproduction. For instance, a recent investigation conducted by Qiang et al. [133] involved a 60-day feeding experiment to evaluate the effects of dietary astaxanthin on the reproductive aspects of female Nile tilapia. The experiment incorporated different levels of astaxanthin in the diets: 0 mg/kg (control), 50 mg/kg, 100 mg/kg, 150 mg/kg, and 200 mg/kg. Notably, the group of fish fed with 150 mg/kg astaxanthin exhibited considerable enhancements in terms of growth, feed utilization, as well as viscerosomatic and hepatosomatic indices. Gonad development saw improvements with astaxanthin levels of 100 mg/kg and 150 mg/kg, leading to increased gonadosomatic index and larger egg diameter (*p* < 0.05). Ovaries from these groups displayed advanced development, uniform gray-yellow eggs, and marked oocyte growth. Furthermore, higher levels of serum 17 β-estradiol, follicle-stimulating hormone, and luteinizing hormone were observed within these groups (Figure 6). The 150 mg/kg group showcased enhanced gene expression related to hormone receptors, heightened catalase activity, decreased malondialdehyde content, reduced apoptosis, and fewer instances of follicular atresia (*p* < 0.05). Through gene ontology analysis, genes associated with cell division and the cell cycle were found to be enriched. Astaxanthin at a dosage of 150 mg/kg was found to activate follicle development via the inhibition of MAPK signaling, thus enhancing oocyte meiosis and gene expression related to progesterone-mediated maturation (*p* < 0.05). To sum up, the extensive outcomes of this study highlight the potential of astaxanthin supplementation to intricately modulate various physiological mechanisms underlying fish reproduction, offering a promising avenue for further research in the field of aquatic reproductive biology.

Gamete quality significantly influences the success of fertilization and subsequent embryonic development in fish. The beneficial impact of astaxanthin on gamete quality has been substantiated by multiple scientific investigations [134,135]. The principal mechanism underlying this enhancement is attributed to astaxanthin’s potent antioxidant properties. The metabolic intensity during gametogenesis renders fish gametes susceptible to oxidative stress [136,137]. By effectively neutralizing ROS, astaxanthin acts as a safeguard, shielding gametes from oxidative damage and upholding their functionality and viability.

Ahmadi et al. [134] conducted a comprehensive inquiry into the effects of astaxanthin supplementation in the diets of female and male rainbow trout broodstock over a controlled 6-month period with regulated lighting conditions. The experiment involved five distinct groups of females, each exposed to diets containing varying concentrations of astaxanthin, while two groups of males were subjected to differing levels of astaxanthin supplementation. Female broodstock eggs were divided into two sets and fertilized using sperm from males that had been fed distinct astaxanthin diets. The analyses revealed astaxanthin levels in eggs spanning from 2.03 to 29.79 mg/kg. The supplementation of astaxanthin exhibited positive repercussions on reproductive traits. Notable differentials were observed in parameters such as fertilization rates, the proportion of eggs exhibiting eye pigmentation and those that hatched, as well as the mortality rate of developed embryos (*p* < 0.05). However, no significant disparity was noted in pre-hatch mortality (*p* > 0.05). Male cohorts exposed to varying astaxanthin concentrations also displayed significant differences in fertilization rates. Moreover, a direct association was established between the content of astaxanthin within eggs and key parameters such as fertilization rate, percentage of eggs with developed eyes, and hatching rate (*p* < 0.05). In essence, the inclusion of dietary astaxanthin supplements is an indispensable strategy for optimizing the reproductive performance of rainbow trout.

Moreover, astaxanthin has demonstrated associations with heightened spawning success across diverse fish species [138,139]. The spawning process is a pivotal event within the reproductive cycle, heavily influenced by multifaceted factors encompassing environmental circumstances, hormonal equilibrium, and gamete quality. Notably, astaxanthin’s favorable influence on gamete quality may engender more efficacious fertilization occurrences, subsequently augmenting the probability of viable embryo formation [135,140]. 

In the investigation by Verakunpiriya et al. [138], the effects of diverse concentrations of synthetic astaxanthin (0, 20, 30, and 40 ppm) incorporated into soft-dry pellets (SDP) were assessed in relation to egg quality and spawning performance among yellowtail fish. The experimental subjects, three-year-old specimens with an average mass of 6.1 kg, were independently fed these diets within controlled enclosures for approximately 5 months prior to the spawning phase. Subsequently, groups of seven males and seven females from each dietary cohort were transferred to controlled indoor spawning tanks, facilitating both natural spawning processes and artificial insemination procedures. To induce spawning, both mature cohorts received injections of human chorionic gonadotropin (600 IU/kg fish). Notably, the most favorable outcomes concerning overall production, egg quality, and the count of normally developing larvae manifested among fish nourished with SDP containing 30 ppm astaxanthin (*p* < 0.05). This held true for both the naturally spawning group and the artificially inseminated group. Consequently, it can be inferred that a supplementation level of approximately 30 ppm of astaxanthin within SDP emerges as an optimal choice for heightening the reproductive efficacy of broodstock yellowtail. The outcome is reflected in enhanced egg quality and a heightened likelihood of successful spawning events.

Furthermore, the potential impact of astaxanthin on the regulation of hormone synthesis and activity presents a compelling avenue for explaining its observed influence on spawning success. Notably, studies conducted on fish have indicated that the supplementation of astaxanthin can lead to the upregulation of crucial reproductive hormones such as estradiol, follicle-stimulating hormone, and luteinizing hormone [133]. These hormones play integral roles in the intricate mechanisms of the reproductive system. Consequently, this hormonal modulation has been suggested to enhance the synchronization of spawning events, ultimately culminating in enhanced reproductive success.

An equally noteworthy area of investigation pertains to astaxanthin’s impact on the embryonic development phase in fish reproduction. The embryonic stage is characterized by heightened susceptibility to oxidative stress, a factor that can precipitate developmental anomalies and reduced viability [141,142]. Herein, the antioxidative properties of astaxanthin come to the fore as they assume a pivotal role in mitigating oxidative stress during this critical developmental window. For instance, empirical evidence reveals a positive correlation between elevated astaxanthin levels within eggs and favorable hatching rates in species such as rainbow trout and carp [134,143]. This concurrence strongly implies that astaxanthin supplementation serves a dual purpose: safeguarding embryos against oxidative stress while simultaneously fostering optimal embryonic progression.

The profound implications of astaxanthin extend to the realm of larval survival and early life-stage performance, an area that has garnered substantial scientific attention [144]. The survival of larval organisms holds pivotal importance, exerting notable influence on recruitment dynamics and population sustainability across diverse fish species [145]. Astaxanthin’s contribution to bolstering larval survival can be attributed to its multifaceted effects, among which its role in enhancing immune responses stands out [146]. Moreover, a wealth of empirical data underscores astaxanthin’s capacity to modulate the expression of immune-related genes in fish larvae, thereby engendering heightened overall survival rates [147,148]. Table 4 presents the effects of astaxanthin on reproduction and larval survival across various fish species.

## 7. Practical Applications in Aquaculture

The incorporation of astaxanthin into aquaculture practices holds significant potential across various aspects, as supported by scientific studies. Notable areas of impact include enhancement of pigmentation [10], immune modulation [146], stress alleviation [107], reproductive performance [133,154], and overall fish health [135,155]. This integration aligns with both economic and ecological objectives, presenting the prospect of diminishing reliance on conventional antibiotics and promoting sustainable growth within the aquaculture industry [157].

Astaxanthin’s role as a potent antioxidant is pivotal in fostering fish well-being within aquaculture systems [155,158]. By incorporating astaxanthin-rich diets for fish, their antioxidant defenses can be reinforced, countering the adverse effects of oxidative stress [5,96,98]. This aspect is particularly pertinent in intensively managed aquaculture settings where high stocking densities and suboptimal environmental conditions often give rise to heightened oxidative stress levels in fish populations [159]. Additionally, astaxanthin’s ability to enhance the immune system can enhance fish disease resistance, thereby reducing susceptibility to prevalent pathogens [5,160]. Through augmentation of innate immune responses, astaxanthin supplementation presents a viable and sustainable strategy for managing diseases in aquaculture, potentially curbing the necessity for prophylactic antimicrobial usage.

Beyond its physiological benefits, astaxanthin holds promise for enhancing the visual appeal of aquaculture products through scientifically supported mechanisms [158]. The modulation of fish pigmentation, a trait of great interest to consumers, can be achieved by integrating astaxanthin into their dietary regimen with demonstrable effects [10]. Valuable fish species like salmon and trout, recognized for their vibrant red or pink coloration, owe this characteristic to dietary astaxanthin content [161]. This attribute not only bolsters the marketability of aquaculture products but also serves as a reliable indicator of fish well-being and vitality [162]. Astaxanthin-enriched diets not only enhance fish coloration but also extend its duration, ensuring a prolonged period of market desirability [163], thereby underscoring the substantial economic implications of astaxanthin utilization within aquaculture, where enhanced pigmentation can command premium prices.

Multiple empirical cases and successful instances underscore the pragmatic feasibility of incorporating astaxanthin into aquaculture practices [5,10,23,134]. In the context of salmonid aquaculture, particularly in regions like Norway and Chile, the longstanding practice of astaxanthin supplementation has been integral to ensuring optimal pigmentation and overall fish health [161,164]. These case studies have effectively showcased that a precisely tailored astaxanthin-rich diet can yield superior coloration while also conferring notable health advantages. Beyond salmonids, other commercially valuable species such as shrimp and ornamental fish have exhibited enhanced reproductive performance upon astaxanthin supplementation, marked by augmented gonadal size, improved gonadosomatic indices, superior egg and semen quality, and enhanced overall reproductive success [135,154,165]. These findings solidify astaxanthin’s role in advancing aquaculture productivity.

Furthermore, the impact of astaxanthin on fish reproduction goes beyond mere enhancement of egg quality, encompassing a broader range of effects. Emerging research suggests that astaxanthin possesses the capability to modulate intricate hormonal pathways, particularly those associated with reproductive processes [133]. This regulatory activity has the potential to induce heightened spawning frequency, increased egg production, and enhanced survival rates in larvae. These discoveries offer promising avenues for refining aquaculture methodologies aimed at optimizing reproductive yields, thereby facilitating the sustainable advancement of the industry.

The incorporation of astaxanthin into aquaculture practices aligns seamlessly with the prevailing emphasis on sustainable approaches, mitigating the reliance on bacteriostatic agents [157]. This integration exemplifies a strategic initiative that harmonizes with the essential goal of diminishing antimicrobial drug employment within aquaculture. By bolstering immune mechanisms and augmenting disease resistance in aquatic species, astaxanthin underscores a comprehensive approach to ecologically conscious aquaculture.

The optimal dosage of astaxanthin in fish diets can fluctuate based on multiple variables, such as the fish species, their developmental stage, size, and the specific objectives, like improving pigmentation or supporting overall health [166]. Numerous researchers posit that astaxanthin may serve as a critical dietary component in aquatic nutrition, warranting its inclusion in diets at concentrations of a minimum of 10 mg/kg [167]. To optimize pigmentation in salmonids, recommended dosages of synthetic astaxanthin can vary based on several factors. These factors include the absence or presence of preharvest assessments, the specific fish species, the desired coloration intensity, the product’s form (such as beadlet or cold-water dispersible formulation), the bioavailability of the formulated product, and various other considerations [168]. Typical dosage ranges normally fall between 40 and 90 mg/kg of feed. There is also evidence suggesting that higher supplementation levels of astaxanthin, reaching up to 200 ppm or even higher, may confer health and reproductive benefits to various fish species [166]. However, it is crucial to note that when using astaxanthin in practice, adherence to local legal regulations is imperative. Therefore, any application of astaxanthin must be carried out in accordance with the applicable legal provisions. For example, within the European Union, EFSA [169] deemed synthetic astaxanthin safe for salmonids at levels of up to 100 mg/kg in their complete diet. This establishes a limit on the additional application of this carotenoid beyond the allowed inclusion level.

Finally, despite extensive research into the positive impacts of astaxanthin on aquatic animals, a comprehensive assessment or documentation of its adverse effects and potential toxicity remains notably lacking [166]. In accordance with EFSA [169], synthetic astaxanthin exhibited excellent tolerance among salmonids and ornamental fish, even at levels as high as 0.91 g/kg. This tolerance significantly surpasses the evaluated levels of this carotenoid in the studies presented within this review. Consequently, it becomes imperative to conduct further research to explore the impact of elevated astaxanthin levels across various fish species.

## 8. Future Directions and Research Gaps

As discussed in this review, the intricate interplay between astaxanthin, the mitigation of oxidative stress, and its potential effects on reproductive performance has been methodically investigated. From its role in enhancing pigmentation to its pronounced antioxidant capabilities, astaxanthin has demonstrated the capacity to enhance not only the visual appeal of aquaculture products but also the overall health and vigor of cultivated fish [157,170]. As we wrap up this comprehensive examination, the fusion of scientific inquiry and pragmatic application prompts the aquaculture sector to delve deeper into the nuanced dimensions of how astaxanthin influences oxidative stress responses and reproductive dynamics. Through ongoing research endeavors, we are positioned to enrich our understanding of astaxanthin’s effects and leverage its benefits to drive sustainable expansion and resilience within global aquaculture practices.

With the continuous growth of the aquaculture industry to meet escalating global seafood demand, there arises an imperative to explore novel strategies that can optimize production efficiency, enhance product quality, and ensure the enduring sustainability of aquaculture methodologies [171,172]. Astaxanthin, celebrated for its robust antioxidant and pigmentation attributes, has surfaced as a promising agent to address these imperatives. However, despite significant strides in comprehending the favorable impacts of astaxanthin supplementation across diverse aquaculture species, numerous pivotal areas of research and forthcoming trajectories remain uncharted.

One notable avenue of research focuses on identifying specific fish species that could derive substantial benefits from astaxanthin supplementation. While numerous studies have underscored the favorable influence of astaxanthin on growth, pigmentation, and immune response across diverse species such as salmon, trout, shrimp, and crustaceans, it is important to note that these effects are subject to species-specific variations [166,173]. Examining the distinct responses of various fish species to astaxanthin supplementation in terms of growth performance, immune function, and overall health holds promise for revealing the range of species that can gain the most from this supplementation. Conducting such investigations would necessitate comprehensive analyses spanning physiology, biochemistry, and molecular mechanisms to unveil the underlying reasons for the observed effects [157]. 

Furthermore, a critical frontier for further research lies in determining the optimal astaxanthin dosage for different species [169]. The relationship between dosage and response is intricate and influenced by factors like fish size and species, dietary composition, intestinal absorption, metabolism and excretion, environmental factors, and life stage [174,175]. A robust grasp of the ideal astaxanthin dosage can serve to mitigate the risk of excessive supplementation, which could result in unfavorable effects or unnecessary economic burdens [10,169]. Through well-designed investigations into dose-response patterns, researchers can establish dosage recommendations customized to specific aquaculture species, encompassing both near-term growth enhancements and enduring health advantages.

The exploration of the extended implications resulting from astaxanthin supplementation demands a comprehensive inquiry. Although temporary enhancements in growth rate, pigmentation, and immune response have been documented over shorter periods [110,157], a deeper understanding of the potential repercussions of prolonged astaxanthin exposure on fish physiology and metabolism is warranted. Extensive investigations into the enduring effects can elucidate the cumulative impact of astaxanthin on fish well-being, reproductive dynamics, and even potential ramifications on product quality. The assessment of astaxanthin compound stability throughout the production cycle and its possible transfer to consumers through the food chain holds paramount significance [110]. By addressing these long-term ramifications, the aquaculture sector can ensure the responsible application of astaxanthin and its sustainable integration into production methodologies.

In the pursuit of unraveling the molecular mechanisms underpinning astaxanthin’s effects in aquaculture, the incorporation of advanced methodologies such as omics studies exhibits substantial promise [176]. The emergence of genomics, transcriptomics, proteomics, and metabolomics has transformed our capacity to scrutinize intricate biological processes at a molecular level [177,178]. The fusion of these omics approaches with conventional physiological and biochemical analyses can yield a comprehensive comprehension of how astaxanthin shapes gene expression, protein synthesis, and metabolic pathways in aquaculture species. For instance, transcriptomic investigations can uncover the gene networks linked with growth promotion, immune modulation, and antioxidant defense in response to astaxanthin supplementation [179,180]. Proteomic assessments can pinpoint specific proteins subject to upregulation or downregulation upon astaxanthin exposure, offering insight into the pivotal functional contributors involved in the observed effects [181]. Metabolomic profiling can divulge insights into the metabolic shifts prompted by astaxanthin and unearth potential indicators of its advantageous outcomes [182]. 

Moreover, the implementation of omics studies can facilitate an all-encompassing evaluation of the interplay between astaxanthin and other dietary constituents. Nutrient interactions exert a pivotal influence on determining the overall efficacy of astaxanthin supplementation [183]. Through the application of systems biology approaches, researchers can delve into how astaxanthin modulates pathways connected to nutrient uptake, utilization, and metabolism. This integrative methodology can yield a more accurate portrayal of the intricate networks governing the physiological responses to astaxanthin and guide the development of optimized diets for aquaculture species.

## 9. Conclusions

The delicate equilibrium between ROS generation and antioxidant defenses plays a central role in maintaining the health of fish. When susceptibility to oxidative stress prevails, it can result in cellular harm, DNA mutations, and disruption of crucial biomolecules. ROS in fish originate from cellular respiration, peroxisomal reactions, inflammation, and environmental contaminants, necessitating robust mitigation approaches.

Astaxanthin, a potent antioxidant, offers a promising solution to counter the adverse effects of oxidative stress on fish. Its unique molecular structure enables interactions with ROS and seamless integration into cell membranes, effectively curbing lipid peroxidation. Moreover, astaxanthin’s positive impact on fish reproduction, including enhanced gamete quality, embryonic development, and hormonal pathways, underscores its versatility. Within aquaculture, astaxanthin provides an array of benefits, from bolstering pigmentation and immune support to reducing stress and improving reproductive outcomes, aligning with economic and ecological objectives. Its successful application across various species highlights its potential to address critical aspects of fish health.

Future research should delve into species-specific advantages, optimal dosage determination, and long-term effects on physiology and product quality. Advanced omics methodologies can unveil the intricate molecular mechanisms underlying astaxanthin’s effects, enhancing our understanding of its role in promoting fish health and fostering sustainable aquaculture growth.

In summary, integrating astaxanthin into aquaculture holds significant promise for enhancing fish well-being, reproductive success, and industry sustainability, offering a responsible and holistic approach to aquaculture development. Acknowledging the nuanced connection between oxidative stress and fish health becomes imperative for effective conservation and management approaches for these vital species as humanity’s influence on aquatic environments continues to escalate.

## Figures and Tables

**Table 1:** Some common oxidative stressors for fish and their permissible limits.

Stressor	Permissible Limit	Effects on Fish	Reference
Oxygen depletion	Dissolved oxygen levels should not fall below 5 mg/L for most freshwater fish.	Low dissolved oxygen can lead to fish suffocation and reduced growth.	[66]
Temperature fluctuations	Diurnal fluctuations in water temperature should not exceed a certain threshold, which is species-specific.	Rapid temperature changes can stress fish and impact their metabolism.	[67]
Pollutants (heavy metals)	Varies by metal and species. In general, allowable concentrations are low (micrograms per liter or lower).	Heavy metals like lead, mercury, and cadmium can accumulate in fish tissues and harm health.	[68]
Pesticides and herbicides	Varies by chemical and species. Generally, very low concentrations are allowed (parts per billion).	These chemicals can disrupt fish physiology and impair reproduction.	[69]
Ammonia	Total ammonia nitrogen levels should be below 0.02 mg/L for freshwater fish.	High ammonia can damage fish gills and cause respiratory distress.	[70]
pH	Optimal pH ranges from 6.5 to 9.0, depending on the fish species.	Extreme pH levels can stress fish, affecting ion balance and survival.	[71]
Salinity	Varies widely by fish species. Some tolerate freshwater, while others require high salinity.	Salinity outside a fish’s tolerance range can cause osmotic stress.	[72]
UV radiation	Exposure should be limited, especially in shallow, clear waters.	Prolonged UV exposure can damage fish skin and eyes.	[73]
Microorganisms (pathogens)	The presence of pathogens like bacteria, viruses, and parasites should be minimized.	Infections can weaken fish and lead to disease outbreaks.	[74]
Toxic algal blooms	Concentrations of harmful algae should be monitored and controlled.	Toxins produced by algae can harm fish and other aquatic organisms.	[75]

**Table 2:** Hepatic antioxidant statuses of golden pompano-fed diets with and without supplementation of astaxanthin [98].

	T-AOC (U/mg Protein)	SOD (U/mg Protein)	GSH (μmol/g Protein)
Diet 1	0.11 ± 0.01 ^a^	240.87 ± 5.76 ^a^	82.44 ± 4.87 ^a^
Diet 2	0.15 ± 0.01 ^b^	214.24 ± 5.71 ^b^	118.52 ± 8.93 ^b^

Values are the mean ± SEM of four replicates, and values in the same column with different letters are significantly different (*p* < 0.05). Diet 1 = fish fed diets without supplementation with astaxanthin. Diet 2 = fish with supplementation of astaxanthin. T-AOC = total antioxidant capacity; GSH = reduced glutathione; and SOD = superoxide dismutase.

**Table 3:** Effects of astaxanthin on oxidative status and growth across various fish species ^1^.

Fish Species	Astaxanthin Supplementation Levels in Feed	Form	Challenge ^2^	Astaxanthin Effects on Oxidative Status	Astaxanthin Effects on Growth	Reference
Rainbow trout (*Oncorhynchus mykiss*)	0.5, 2.0 g/kg	Synthetic	Yes	Reduced MDA and peroxide values and upregulation of the expression of antioxidant-relevant genes in fish fillet	Improved final weight and FCR	[97]
Yellow catfish (*Pelteobagrus fulvidraco*)	0.08 g/kg	Synthetic	Yes	Higher levels of catalase activity and reduced MDA in the liver	Improved final weight and specific growth rate	[107]
Rainbow trout (*Oncorhynchus mykiss*)	0.5, 2.0, 5.0 g/kg	Synthetic	Yes	Reduced MDA and improved T-AOC in blood serum, upregulation of the expression of antioxidant-relevant genes in the liver	Improved final weight and specific growth rate	[23]
Characin (*Hyphessobrycon eques* Stein-dachner)	0.01, 0.02, 0.04 g/kg	Synthetic	Yes	Improved antioxidant capacity as measured by total antioxidant status and superoxide dismutase activity	No effect	[108]
Rainbow trout (*Oncorhynchus mykiss*)	0.1 g/kg	Synthetic	Yes	Decrease in TBARS in muscle and liver cells; increased glutathione reductase activity; improved ratio of GSH to GSSG	Numerical improvement in final weight	[96]
Discus fish (*Symphysodon aequifasciatus*)	0.2 g/kg	Synthetic	Yes	Improved antioxidant defense status	No effect	[109]
Coral trout (*Plectropomus leopardus*)	0.05, 0.1, 0.2 g/kg	Natural	No	Elevated levels of catalase, superoxide dismutase, and glutathione peroxidase activities increased T-AOC in the serum and liver	No effect	[5]
Golden pompano (*Trachinotus ovatus*)	0.2 g/kg	Synthetic	No	Elevated hepatic T-AOC and augmented levels of GSH to GSSG	Improved weight gain, specific growth rate and FCR	[98]
Rainbow trout (*Oncorhynchus mykiss*)	0.1 g/kg	Synthetic and natural	No	IncreasedNrf2/HO-1 signaling and antioxidant enzyme activity	Improved final body weight and FCR	[110]

^1^ Please note that these effects may vary depending on factors such as dosage, fish species, duration of supplementation, type of challenge, and individual fish physiology; ^2^ induced oxidative stress; MDA = malondialdehyde; T-AOC = Total antioxidant capacity; TBARS = Thiobarbituric acid-reactive substances; GSH = Reduced glutathione; GSSG = Oxidized glutathione; FCR = feed conversion ratio; Nrf2 = Nuclear factor erythroid-2 related factor 2; HO-1 = heme oxygenase-1.

**Table 4:** Effects of astaxanthin on reproduction criteria and larval survival across various fish species ^1^.

Fish Species	Astaxanthin Supplementation Levels in Feed	Form	Astaxanthin Effects on Reproduction and Larval Survival Criteria	Reference
Clownfish (*Amphiprion ocellaris*)	0.05, 0.1, 0.15, 0.2	Synthetic	Improved hatching rate of eggs, reduced malformed rate, and increased survival rate of larvae in 3 days post-hatch	[144]
Swordtail fish (*Xiphophorus helleri*)	0.05, 0.1 and 0.2	Synthetic	Improved reproductive parameters	[149]
Nile tilapia (*Oreochromis niloticus*)	0.05, 0.1, 0.15 and 0.2 g/kg	Natural	Improved gonad development, higher levels of serum E2, FSH, and LH, reduced apoptosis, and fewer instances of follicular atresia	[133]
Goldfish (*Carassius auratus*)	0.05, 0.1, 0.15 g/kg	Synthetic	Improved osmolality, motility, spermatocrit value, sperm concentration, and fertilization rate	[135]
Rainbow trout (*Oncorhynchus mykiss*)	0.07, 12.5, 33.3, 65.1 or 92.9 mg/kg	Synthetic	Improved fertilization rates, the proportion of eggs exhibiting eye pigmentation and those that hatched, as well as the reduced mortality rate of developed embryos	[134]
Yellowtail (*Seriola quinqueradiata*)	0.02, 0.03, 0.04 g/kg	Synthetic	Improved fertilization rate, egg quality, hatching rate, and the count of normally developing larvae	[138]
Atlantic cod (*Gadus morhua* L.)	0.1 g/kg	Synthetic	Lower egg incubation mortality and higher larval growth and survival	[139]
Gold fish (*Carassius auratus*)	0.05, 0.1, 0.15 g/kg	Synthetic	Higher number of hatched eggs, larvae produced, and survivability	[150]
Fighter fish (*Betta splendens*)	0.05, 0.1, 0.15 g/kg	Synthetic	Higher hatchability and a higher survival rate of larvae	[151]
Sea bream (*Sparus aurata*)	“Enriched in astaxanthin”	Natural	Higher survival rate of larvae	[152]
Clownfish (*Amphiprion clarkia*)	“Enriched in astaxanthin”	Natural	Improved development and survival of larvae	[153]
Goldfish (*Carassius auratus*)	0.05, 0.1, 0.15 g/kg	Synthetic	Higher diameter and number of eggs per gram of fertilized eggs result in higher egg survival rates in the incubation period	[154]
Atlantic Cod (*Gadus morhua* L.)	0.074 g/kg	Synthetic	A higher number of eggs per batch spawned and improved numbers of fertilized eggs per kg of female	[155]
Striped Jack (*Pseudocaranx dentex*)	0.01 g/kg	Synthetic	Improved overall spawning performance	[156]

^1^ Please note that these effects may vary depending on factors such as dosage, fish species, duration of supplementation, type of challenge, and individual fish physiology. LH = luteinizing hormone; FSH = follicle-stimulating hormone; E2 = estradiol.

## Data Availability

Not applicable.

## Abbreviations

DNA	Deoxyribonucleic acid
E_2_	Estradiol
FCR	Feed conversion ratio
FSH	Follicle-stimulating hormone
H_2_O_2_	Hydrogen peroxide
IU	International Unit
LH	Luteinizing hormone
MAPKs	Mitogen-activated protein kinases
Nrf2	Nuclear factor erythroid-2 related factor 2
NF-κB	Nuclear factor-kappa B
GSSG	Oxidized glutathione
ROS	Reactive oxygen species
GSH	Reduced glutathione
NADPH	Reduced nicotinamide adenine dinucleotide phosphate
^1^O_2_	Singlet oxygen
SDP	Soft-dry pellets
SOD	Superoxide dismutase
TBARS	Thiobarbituric acid-reactive substances
T-AOC	Total antioxidant capacity
